# Dose of Incorporated Immunodominant Antigen in Recombinant BCG Impacts Modestly on Th1 Immune Response and Protective Efficiency against *Mycobacterium tuberculosis* in Mice

**DOI:** 10.1155/2014/196124

**Published:** 2014-07-23

**Authors:** Hui Ma, Kang Wu, Fang Liu, Hua Yang, Han Kang, Ning-Ning Chen, Qin Yuan, Wen-Jiang Zhou, Xiao-Yong Fan

**Affiliations:** ^1^Shanghai Public Health Clinical Center Affiliated to Fudan University, Shanghai 201508, China; ^2^Key Laboratory of Medical Molecular Virology of MOE/MOH, Shanghai Medical College, Fudan University, Shanghai 200032, China

## Abstract

One approach for improving BCG efficacy is to utilize BCG as vehicle to develop recombinant BCG (rBCG) strains overexpressing *Mycobacterium tuberculosis (M. tb)* antigens. Also expression level of a candidate antigen should impact the final T cell responses conferred by rBCG. In this study, based on our previously constructed differential expression system, we developed two rBCG strains overexpressing *M. tb* chimeric antigen Ag856A2 (coding a recombinant *ag85a* with 2 copies of *esat-6* inserted at *Acc* I site of *ag85a*) at differential levels under the control of the subtly modified *furA* promoters. These two rBCG strains were used to vaccinate C57BL/6 mice and exploit dose of incorporated antigen in rBCG to optimize immune response and protective efficiency against *M. tb* challenge in mouse model. The results showed that rBCG strains overexpressing Ag856A2 at differential levels induced different antigen-specific IFN-*γ* production and comparable number of *M. tb*-specific CD4 T cells expressing IL-2. *M. tb* challenge experiment showed that rBCG strains afforded enhanced but comparable immune protection characterized by reduced bacillary load, lung pathology, and inflammation. These results suggested that the dose of antigens incorporated in rBCG can impact T cell immune responses but imposed no significantly differential protective efficacies.

## 1. Introduction

Tuberculosis (TB) caused by* Mycobacterium tuberculosis (M. tb)* continues to be a significant global health problem, affecting millions of people worldwide [1, 2]. Approximately 95% of all TB cases occur in the developing world [3]. It is a prevalent infectious disease in China, with 250,000 deaths from TB annually and 6 million active TB patients at present [4]. The global incidence of TB is raising due to coinfection with the human immunodeficiency virus (HIV) and the emergence of multidrug-resistant (MDR)* M. tb* strains [5, 6]. According to the report of World Health Organization (WHO),* M. tb* will cause 1 billion new cases and about 35 million deaths worldwide by 2020 [7]. Therefore, effective treatment and control strategies are urgently needed to counteract the global threat of TB.

The current vaccine against TB,* M. bovis* Bacilli-Calmette-Guérin (BCG), is a live attenuated vaccine which has been widely used throughout the world for many decades. BCG protects children efficiently against miliary and meningeal TB, but protective efficiency against pulmonary TB in adults has been found to vary highly from 0% to 80% [8]. Much effort has been devoted to improving BCG efficacy by genetic engineering technology because of its strong immunostimulatory properties and proven safety for human use [9, 10]. Recombinant BCG (rBCG) expressing different immunodominant antigens of* M. tb*, such as secreted antigens (Ag85B, Ag85C, ESAT-6, etc.) or latency associated antigens (*α*-Crystallin, Rv2659c, Rv3407 and Rv1733c, etc.), have been tested as candidate vaccines against TB and are demonstrated to have an enhanced ability to induce Th1 immune response and protection against* M. tb* challenge in animal models [11, 12]. Also, it is definitely no doubt that doses of antigens could subtly influence the magnitude of host immune response as well as protection efficacy, no matter antigen is administered in the form of rBCG [13], protein [14], DNA [15], or RNA [16].

We have previously reported the construction of a* M. tb furA* gene operator/promoter (p*furA*)-based differential expression system, from which it is feasible to express target antigens of interest in a modular fashion [4]. This system will facilitate the development of novel recombinant BCG vaccine candidates.* M. tb* chimeric antigen Ag856A2, which is coded by a recombinant* ag85a* gene with 2 copies of* esat-6* gene inserted at the* Acc* I site of* ag85a* (see Figure S1 in Supplementary Material available online at http://dx.doi.org/10.1155/2014/196124), shows improved immunogenicity in mice when it is inoculated intramuscularly as a DNA vaccine [17]. For the current study, we selected two rBCG strains overexpressing the same chimeric antigen Ag856A2 at the maximum difference: rBCG186 and rBCG486 overexpressing the fusion protein under control of the wild-type or the optimized double-mutated* furA* promoters, respectively [4]. We tested their efficacy as vaccines in C57BL/6 mice, comparing immune response and protection against* M. tb* challenge. The results showed that mice vaccinated with rBCG186 or rBCG486 generally induced higher antigen-specific effector and memory immune responses, as well as protective efficacies compared to mice vaccinated with the parent BCG strain. However, the two rBCG strains between themselves, which expressed the chimeric antigen Ag856A2 at different levels, induced different antigen-specific IFN-*γ* production and comparable number of* M. tb*-specific CD4 T cells expressing IL-2. And the protective efficacies imposed by the two rBCG strains displayed no significant differences although higher protection was observed in rBCG486 vaccinated mice than that in rBCG186 vaccinated mice.

## 2. Materials and Methods

### 2.1. Experimental Animals and Ethics Statement

Female specific pathogen-free (SPF) C57BL/6 mice aged 6–8 wks were purchased from Shanghai SLAC Laboratory Animal Co., Ltd. (Shanghai, China) and kept under SPF conditions with food and water* ad libitum* until challenge. Infected mice were maintained in a biosafety level 3 (BSL-3) biocontainment animal facility. All animal experiment protocols were approved by Chinese Science Academy Committee on Care and Use of Laboratory Animals and were performed according to the guidelines of the Laboratory Animal Ethical Board of Shanghai Public Health Clinical Center.

### 2.2. Bacterial Strains and Growth Conditions

*E. coli* DH5*α* was cultured in liquid or solid LB medium.* M. bovis* BCG-Danish was kindly gifted from Shanghai Institute of Biological Products. BCG and its derivative recombinant strains were grown in liquid Middlebrook 7H9 broth (BD Difco, USA) supplemented with 10% oleic acid-albumin-dextrose-catalase enrichment (OADC, BD Difco, USA), 0.2% glycerol, and 0.05% Tween 80. Cultures in the exponential phase were frozen and stored at −80°C. When required, kanamycin was added at a final concentration of 50 or 20 *μ*g/mL for* E. coli* or mycobacteria, respectively.

### 2.3. Plasmid Construction and Recombinant BCG Strains Preparation

Two rBCG strains overexpressing* M. tb* chimeric immunodominant antigen Ag856A2 at different levels were constructed as previously described [4]. Briefly, the Ag856A2 coding gene, which is a recombinant* ag85a* gene with 2 copies of* esat-6* gene inserted in its* Acc* I site [17], was amplified from the plasmid template of DNA vaccine HG856A and then cloned into mycobacterial differential expression vectors pMFA11 and pMFA41 under control of the prototypical and double-mutated (mutations: initial codon change from GTG to AUG and 6 bp substitution at upstream AT-rich region)* furA* promoter, respectively. The resulting constructs were electroporated into BCG-Danish competent cells and selected on Middlebrook 7H11 agar with kanamycin. The rBCG transformants were grown to midexponential phase in complete Middlebrook 7H9 broth and then verified the recombinant protein expression by routine Western-blotting assay.

### 2.4. Mouse Immunization and* M. tb* Challenge

Mice were vaccinated subcutaneously (*s.c.*) with 2 × 10^6^ colony-forming units (CFU) of BCG or rBCGs in 100 *μ*L saline. Eight weeks after vaccination, groups of 6 mice were either sacrificed for assessment of antigen-specific T cell responses in splenocytes or exposed to an aerosol of virulent* M. tb* H37Rv strain to deposit an inhaled dose of 100–200 CFU per lung by an inhalation exposure system (Glas-Col, USA) [18].

### 2.5. *Ex Vivo* IFN-*γ* ELISPOT Assay

IFN-*γ* ELISPOT assay kit (BD Biosciences, USA) was used as described by the manufacturer. Plates were coated with anti-IFN-*γ* mAb overnight at 4°C and then blocked with RPMI 1640 medium containing 10% fetal bovine serum (FBS) for 1 h at room temperature. Splenocytes (2.5 × 10^5^ cells/well) from immunized mice were isolated, plated, and cultured with 10 *μ*g/mL PPD (Statens Serum Institute, Denmark) or 2 *μ*g/mL recombinant Ag85A, 6 *μ*g/mL recombinant ESAT-6 to provide stimulation at 37°C, 5% CO_2_ for 20 h. After washing the plates with PBS-T20 (1 × PBS, pH 7.4, 0.05% Tween 20), biotinylated anti-IFN-*γ* was added for 2 h at room temperature. Streptavidin-HRP was added for 45 min, and the color was developed with 3-amino-9-ethylcarbazole (AEC) substrate (BD Biosciences). An immunospot analyzer (Cellular Technology, USA) was used to count the spots.

### 2.6. Flow Cytometric Analysis of Intracellular Cytokine Production

Splenocytes (2 × 10^6^ cells/well) isolated at 8 weeks after immunization were plated in 96-well plates and stimulated with 10 *μ*g/mL PPD for 14 h in the presence of 1 *μ*g/mL anti-CD28 (BD Biosciences) and subsequently incubated for an additional 5 h at 37°C following the addition of 0.5 *μ*L/mL monensin/GolgiStop (BD Biosciences). Following overnight incubation at 4°C, the cells were washed in FACS buffer (PBS containing 0.1% sodium azide and 1% FBS) and subsequently stained for 30 min at 4°C for surface markers with mAbs as indicated using anti-CD3-Pacific Blue, anti-CD8-FITC, and anti-CD44-V500 (all from BD Biosciences). Cells were then washed in FACS buffer, fixed, permeabilized using the Cytofix/Cytoperm kit (BD Biosciences) according to the manufacturer's instructions and stained intracellularly for 30 min at 4°C using anti-IFN-*γ*-APC-Cy7, anti-TNF-*α*-Percp-Cy5.5, and anti-IL-2-APC mAbs (all from BD Biosciences). Cells were subsequently washed, resuspended in FACS buffer, and then analyzed by multiparameter flow cytometry using a BD FACSAria flow cytometer (BD Biosciences). For each sample, at least 300,000 events were collected and responses were analyzed using FlowJo software (Tree Star, USA).

### 2.7. Bacterial CFU Assay

Five weeks after infection, mice were sacrificed and the mycobacterial burden was determined by plating homogenates of lung, excluding right postcaval lobe, and entire spleen onto Middlebrook 7H11 agar plates supplemented with 10% OADC enrichment and a 4-antibiotic mixture (40 U/mL polymycin B, 4 *μ*g/mL amphotericin, 50 *μ*g/mL carbenicillin, 2 *μ*g/mL trimethoprim) that prevents growth of contaminating microorganisms. Plates were incubated at 37°C for 3 weeks in semisealed plastic bags and then CFU were counted and expressed as log_10_ CFU per organ.

### 2.8. Histopathological Analysis

The right postcaval lobes were fixed in formalin and embedded in paraffin. Then, the embedded lung lobes were sectioned in thickness of 5 *μ*m, stained with haematoxylin and eosin (H & E) and photographed using a Olympus CKX41 microscope (Olympus, Japan) fitted with an Olympus DP20 camera connected to a computer. The Image Pro Plus program (Media Cybernetics, USA) was utilized to objectively assess the level of inflammation present in each image. The inflamed areas stained a more intense purple than the noninflamed areas. The mean percent of area inflamed was quantified averaging from three to five lung sections of each of the different groups of mice.

### 2.9. Immunohistochemistry

Immunohistochemistry of lung sections was performed as previously described [19]. The antibodies were rabbit polyclonal anti-mouse TNF-*α* (Abcam, UK), rabbit polyclonal IFN-*γ* antibodies (Invitrogen, USA), and rabbit polyclonal anti-mouse iNOS antibody (Cayman Chemical, USA). All sections were examined by light microscopy, and the expression of TNF-*α*, IFN-*γ*, or iNOS was semiquantified by intensity of positive signal using Image Pro Plus software.

### 2.10. Statistical Analysis

Immune responses, protective efficacies, and histopathological staining were tested by one-way ANOVA followed by Tukey's multiple comparison tests of the means. Immunohistochemistry staining was compared by a nonparametric Mann-Whitney *U* test. ^∗^ *P* < 0.05  ^∗∗^ *P* < 0.01, or ^∗∗∗^ *P* < 0.001.

## 3. Results

### 3.1. rBCG Strains Overexpress Different Levels of Fusion Protein Ag856A2

**Figure 1:**
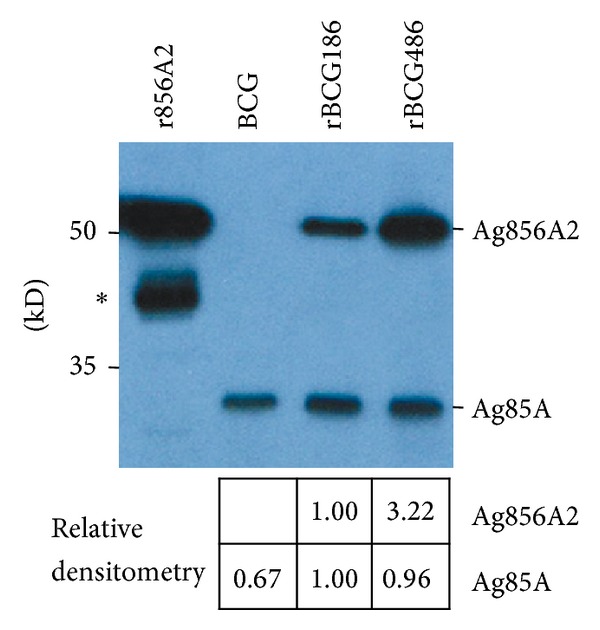
Expression of chimeric antigen Ag856A2 in rBCG strains by Western blotting. Equal amounts of lysates supernatant from cell crude extracts of (r)BCGs were run in SDS-PAGE and then probed with mouse antiserum to Ag85A. The origin of the lysate is marked in the top. Band intensities of Ag856A2 and Ag85A from BCG, rBCG186, and rBCG486 were quantified by densitometry (Image-J software) and divided by cognate band intensity from rBCG186 (relative densitometry). Lane “r856A2” represents rAg856A2 purified after expression in* E. coli* and serving as the positive control. The staining intensity of native Ag85A was roughly at the same level in all lanes, indicating that equal amounts of whole cell lysates were loaded. The result is representative of two independent experiments. ^∗^The degradative form of rAg856A2.

We have previously developed a novel mycobacterial differential expression system (pMFA series) based on the* M. tb furA* gene operator/promoter (p*furA*) or its derivatives. Ag856A2 was cloned into two of these plasmids, pMFA11 and pMFA41, which drives low and high gene expression under the control of the wild-type and modified* fur*A promoters, respectively [4]. By transformation of BCG, we obtained two strains, rBCG186 and rBCG486, which drove correspondingly low and high expression of chimeric immunodominant antigen Ag856A2 (Figure 1, upper panel). Quantification of the band intensities of Western-blot indicated that rBCG486 roughly expressed > 3-fold of Ag856A2 than rBCG186 did (Figure 1, lower panel).

### 3.2. Higher Expression of Ag856A2 in rBCG Strains Induces Higher Antigen-Specific IFN-*γ* Response

**Figure 2:**
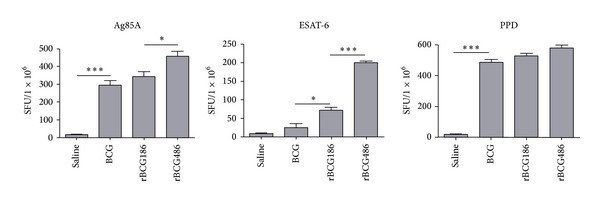
ELISPOT assays of* M. tb*-specific IFN-*γ* producing splenocytes in (r)BCGs-vaccinated mice. Eight weeks after vaccination, splenocytes were isolated and then incubated with purified Ag85A, ESAT-6, or PPD for* ex vivo* IFN-*γ* ELISPOT assay. Column diagram for mean number of spot-forming units (SFU) ± SD (*n* = 6) were shown. ^∗^ *P* < 0.05 or ^∗∗∗^ *P* < 0.001 (one-way ANOVA).

Eight weeks after vaccination, ELISPOT assay of splenocytes showed that more cells in the rBCG486-vaccinated mice expressed Ag85A-specific IFN-*γ* compared to those of rBCG186- and BCG-vaccinated mice (Figure 2, left panel). Also, significantly elevated numbers of splenocytes expressed ESAT-6-specific IFN-*γ* in both rBCG186- and rBCG486-vaccinated mice compared to that of BCG-vaccinated mice (Figure 2, middle panel). Additionally, ESAT-6-specific IFN-*γ* was induced at much higher level in rBCG486-vaccinated mice compared to rBCG186 group (Figure 2, middle panel). A similar pattern of PPD-specific IFN-*γ* responses as Ag85A-specific response was also observed but the difference was not statistically significant, regarding to the comparisons of rBCG486-vaccinated mice and other immunized groups (Figure 2, right panel).

### 3.3. rBCG Vaccination Induce Higher IL-2-Producing CD4 T Cell Responses

**Figure 3:**
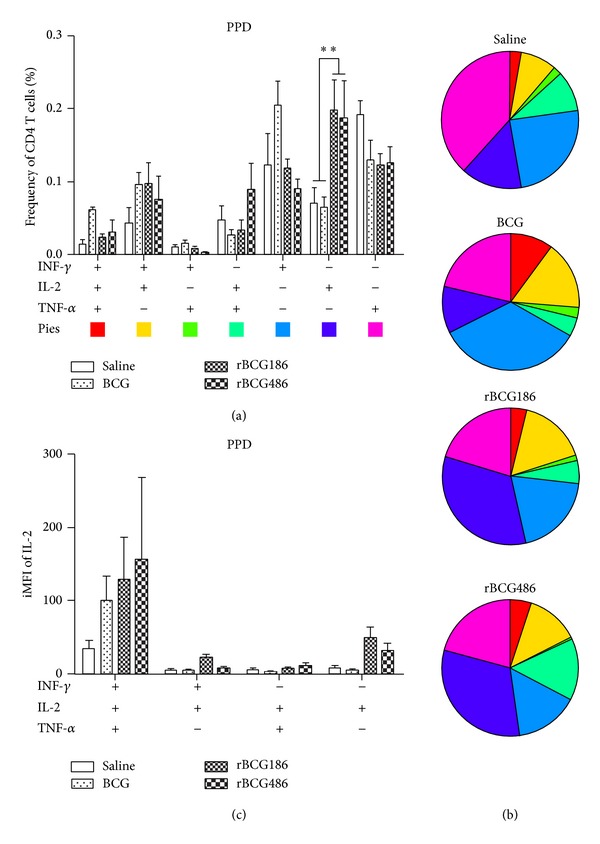
Flow cytometric analysis of intracellular cytokine production after immunization. Eight weeks after vaccination, splenocytes from 6 mice were isolated, pooled and stimulated with PPD for 14 h, and then analyzed for cytokine production by intracellular cytokine staining (ICS) assay. CD3^+^CD4^+^ T cells producing IFN-*γ*, TNF-*α*, and IL-2 were distinguished as seven distinct subpopulations based on their production of these cytokines in any combination. The subpopulation proportions as components of the total CD4^+^ T cell population are shown (a) and their proportions as components of the seven subpopulations are shown in pie chart form (b). Integrated mean fluorescence intensities (iMFI) of IL-2 in four cytokine profiles (c). Data are shown as mean ± SEM. ^∗∗^ *P* < 0.01 (one-way ANOVA).

We used flow cytometry to measure the capacity of* M. tb*-specific CD4 T cells from spleens of vaccinated mice producing cytokines IFN-*γ*, TNF-*α*, and IL-2 at single cell level after stimulation* in vitro* with PPD. The cytokine-producing CD3^+^CD4^+^ cells were classified into seven subpopulations based on their production of IFN-*γ*, TNF-*α*, and IL-2 in any combination (Figure 3(a)).

Significantly increased frequencies of PPD-specific IL-2^+^ CD4 T cells were identified in rBCG-vaccinated mice, whereas increased frequencies of IFN-*γ* ^+^ cells were identified in BCG-vaccinated mice even though statistically insignificant (Figure 3(a)). The pie chart of this data clarified the dominance of IL-2^+^ CD4 T cells in rBCG-vaccinated mice, while IFN-*γ* ^+^ CD4 T cells dominated the responses of BCG-vaccinated mice (Figure 3(b)). rBCG and BCG-vaccination did not differ in their ability to induce* M. tb*-specific CD4 T cells producing other combinations of cytokines (*P* > 0.05). In accordance, we also observed higher integrated mean fluorescence intensities (iMFI = %frequency × MFI) of IL-2 in IL-2-producing CD4 T cells, even though it is statistically insignificant (Figure 3(c)).

### 3.4. Enhanced Protection Conferred by rBCG Vaccination

**Figure 4:**
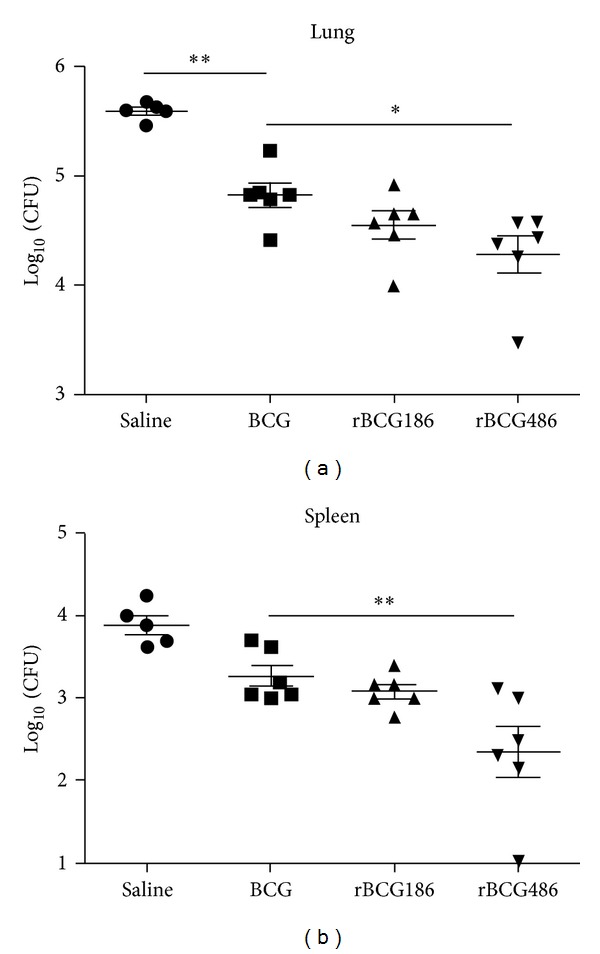
Enhanced protection against* M. tb* challenge by rBCGs vaccination. Eight weeks after vaccination, mice (*n* = 5 or 6) were challenged with* M. tb* H37Rv; bacillary loads in lung (a) and spleen (b) were determined at 5 weeks after infection and expressed as Log_10_ CFU per organ. Representative data from one of two experiments were shown. ^∗^ *P* < 0.05 or ^∗∗^ *P* < 0.01 (one-way ANOVA).

In general, rBCG induced higher antigen-specific cytokine responses as compared to BCG (Figures 2 and 3), and rBCG486 induced higher antigen-specific IFN-*γ* response (Figure 2) and comparable frequency of* M. tb*-specific CD4 T cells expressing IL-2 (Figure 3). Then, we further compared the protective efficacies of rBCG486, rBCG186, and BCG against* M. tb*-challenge. As shown in Figure 4(a), 5 weeks after challenge all vaccinated mice had a significantly reduced bacillary load in lungs, when compared to the saline-treated mice. Vaccination with BCG and rBCG186 resulted in a comparable reduction in bacillary load (Figure 4). However, even though rBCG486 vaccination induced a significantly greater protection when compared to the BCG-vaccinated mice, it showed no difference of protection when compared to the rBCG186-vaccinated mice (Figure 4(a)). The bacillary loads in spleens shared the similar pattern as those in lungs, with rBCG486-vaccinated mice having far fewer bacilli when compared to the saline-treated or BCG-vaccinated mice and having comparable bacilli compared to the rBCG186-vaccinated mice (Figure 4(b)).

### 3.5. Reduced Pulmonary Inflammation following rBCG Vaccination

**Figure 5:**
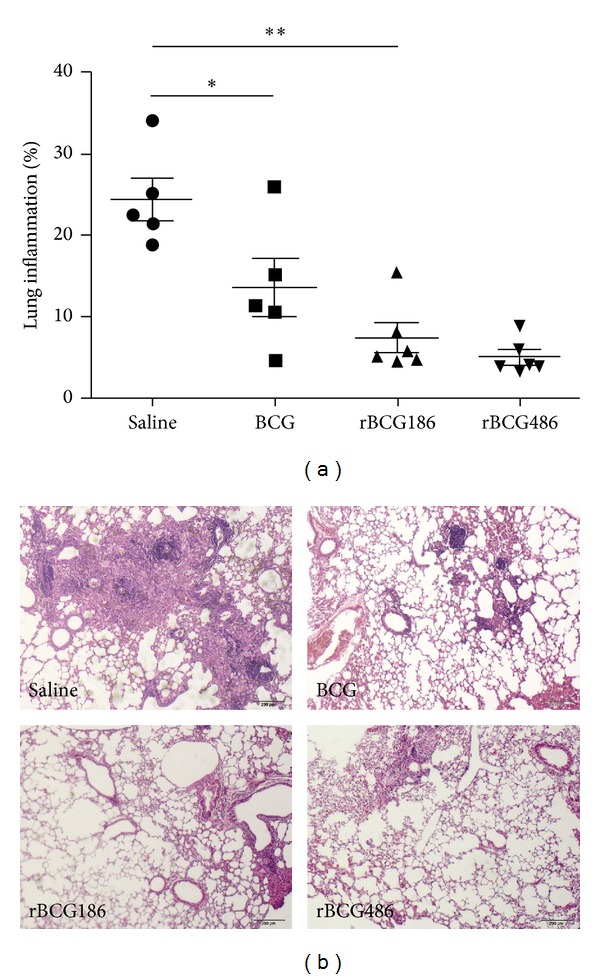
Reduced pathology in vaccinated mice after infection. The percentage of lung area showing infiltration and consolidation was determined by H & E staining 5 weeks after infection (a), and representative histological appearances of lung tissue are shown in the right panel (b). ^∗^ *P* < 0.05 or **P < 0.01 (one-way ANOVA).

Five weeks after challenge,* M. tb* infection caused severe pathology changes in saline-treated mice, with about 24.3% of the tissue showing extensive multifocal granulomatous infiltration, characterized by numerous foamy macrophages surrounded by inflammatory cells (Figure 5). However, all the vaccinated groups of mice had significantly reduced pulmonary granulomatous consolidation compared to the unvaccinated mice (i.e., 13.42% consolidation in BCG-vaccinated group, 7.24% in rBCG186-vaccinated group, and 4.87% in rBCG486-vaccinated group). The rBCG-vaccinated mice showed the mildest pathology, and all of the mice in these two groups had mainly well-preserved alveolar spaces with only a few scattered areas of diffused infiltration (Figure 5).

### 3.6. Localization of TNF-*α*, IFN-*γ*, and Inducible Nitric Oxide Synthase (iNOS) in* M. tb* Infected Lung

**Figure 6:**
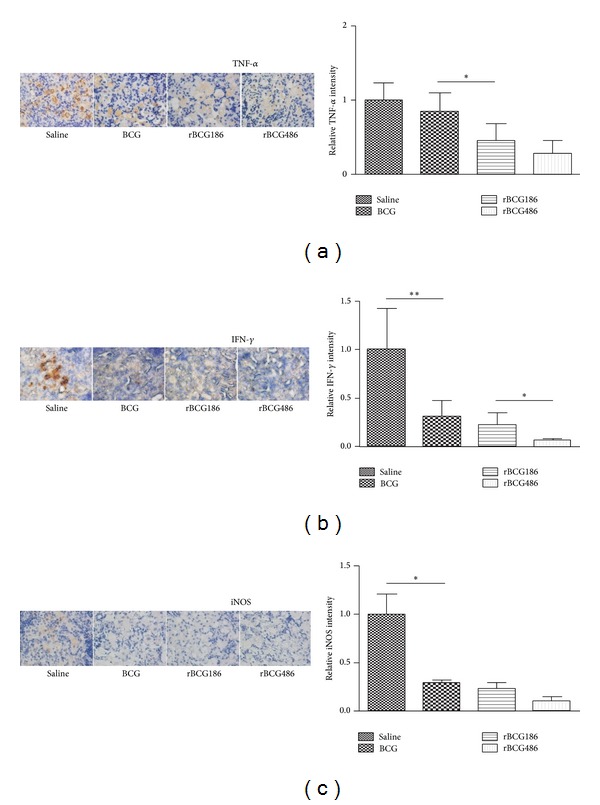
Localization of TNF-*α*, IFN-*γ*, and iNOS in infected lungs of mice 5 weeks after infection. Representative photomicrographs show immunohistochemical staining (brown color) for TNF-*α* (a), IFN-*γ* (b), and iNOS (c) in pulmonary granulomas (left panel). Quantification of staining (intensity × area of staining) is displayed as mean ± SD. ^∗^ *P* < 0.05 or ^∗∗^ *P* < 0.01 (Mann-Whitney *U* test).

Immunohistochemical staining of the lung tissues showed the presence of TNF-*α*, IFN-*γ*, and iNOS in all groups of infected mice and staining was strongest in the granulomatous lesions compared to that in the nongranulomatous areas. However, the extent of staining varied among the groups. Five weeks after infection, a very high level of TNF-*α* was observed in the lungs of saline-treated mice (Figure 6(a)); TNF-*α* staining was extensive in necrotic areas within the advanced coalescent granulomas. Vaccination with BCG resulted in the reduced amounts of TNF-*α* expression, even though statistically insignificant. In contrast, rBCG-vaccinated mice, especially rBCG486-vaccinated mice, showed only a little weak staining for TNF-*α* and this was restricted primarily to the granuloma core (Figure 6(a)). Similar patterns of IFN-*γ* and iNOS staining were also observed except that there was relatively much weaker staining in the lungs of (r)BCG-vaccinated mice compared to the saline-treated mice (Figures 6(b) and 6(c)). Similar pattern of TNF-*α*, IFN-*γ*, and iNOS staining was also observed in the infected spleens of vaccinated mice, with the highest staining in saline-treated mice, moderate staining in BCG-vaccinated mice, and the lowest staining in rBCG-vaccinated mice (Figure  S2).

## 4. Discussion

During the past decades, great efforts have been focused on modifications of the current BCG vaccine to develop new anti-TB vaccine candidates [20]. Some modified rBCG strains, such as rBCG30 and rBCGΔ*UreC*::*Hly*, have been demonstrated to yield improved protection against* M. tb* infection in experimental animal model compared to the existing BCG vaccine and have entered into clinical trial. Nevertheless, it is promising to keep on optimization of BCG protective immune if two points are being issued. One is the fact that the best immunodominant antigen for TB should be precisely defined. Another is that the expression levels of such antigens should be optimal enough to elicit effective immune responses [21]. Here, we constructed two rBCG strains overexpressing immunodominant chimeric antigen Ag856A2 at varying levels depending upon the strengths of the different* furA* promoters [4] and then compared the cellular immune response and protection in mice induced by these two rBCG strains.

One way to improve BCG efficacy is to overexpress mycobacterial immunodominant antigens to induce optimal host immune responses in the life cycle of BCG within host [12, 19]. This kind of strategy reflects that the doses of antigens are one of pivotal factors influencing the protective efficacies of vaccines. Aagaard et al. demonstrated that protective efficiency of TB subunit vaccines is highly dependent on the antigen dose [14]. They vaccinated mice with different doses of fusion protein Ag85B-TB10.4 which were emulsified in adjuvant IC31, and the higher immune response and protective efficacy were only observed when the antigen was administered in proper doses, and decreasing or increasing of the antigen dose would dramatically dwarf the protection efficacies of the antigens [14]. In our study, the cognate antigen Ag856A2 in rBCG186 and rBCG486 was expressed under the control of promoters p*furA* and p*furAma* (Figure S1) [4]. These two promoters, by their nature, were verified to have varied promoter activities, with p*furA* the lower one and p*furAma* the higher one [4], and were consequently used to develop the rBCG strains overexpressing chimeric antigen Ag856A2 at different levels, with lower expression in rBCG186 and higher expression in rBCG486 (Figure 1). And different Ag856A2 antigen loading in rBCGs resulted in differential host immune responses, with the higher antigen-specific effector immune response in the rBCG486-vaccinated mice as validated through* in vitro* IFN-*γ* ELISPOT assay (Figure 2). However, we did not observe the significant differences in the qualities of* M. tb*-specific CD4 T cells coexpressing IFN-*γ*, TNF-*α*, and IL-2 (Figure 3), nor the protection efficacies and lung inflammations, between the two groups of rBCGs-vaccinated mice (Figures 4 and 5). Interestingly, subtly higher percent of polyfunctional CD4 T cells (IFN-*γ* ^+^IL-2^+^TNF-*α* ^+^) was observed in BCG-vaccinated mice compared to other groups of mice (Figure 3(a)); however, the protective efficacy elicited by BCG vaccination is not that effective as rBCGs (Figure 4). This contradictory result could be explained with the fact that the lower iMFI values of IFN-*γ*, IL-2, and TNF-*α* in BCG-vaccinated mice were observed (see the case of IL-2 in Figure 3(c) as representative). MFI provides one measure of the quality of the immune response since the cells that are more actively producing cytokine stain more brightly [22], thus the lower iMFI values of cytokines reflected poor quality although mildly higher frequency of polyfunctional CD4 T cells was seen in BCG-vaccinated mice, and this further emphasizes that not only the magnitude but also the quality of vaccine-induced T cells responses are critical to guide development of effective immunization strategies [23]. In addition, higher IL-2 secretion, both in the levels of percentage and iMFI, were seen in the rBCG486-vaccinated mice than that of BCG group; this data support our recent findings that IL-2 production in the spleens of vaccinated mice after vaccination can predict vaccine efficacy (Kang H, et al. Immunology, 2014; in press). The same explanation might also be used to account for the fact that although the saline-treated mice showed high numbers of cells producing TNF-*α*, the iMFI is relative low (data not shown).

The quality of T cell response has significant effect on the establishment of protective memory [23]. As with the phenotypic heterogeneous nature of T cells, these cells are definitely functional heterogeneous. Thus, in addition to monitoring exclusively the IFN-*γ* response after vaccination, researchers have been focusing on the coexpression of more cytokines at single cell level through flow cytometry technique [24, 25]. The rBCG186 or rBCG486, at least at the time we tested, induced much higher frequencies of IL-2^+^ CD4 T cells responding to PPD stimulation in splenocytes compared to the saline-treated or BCG-vaccinated mice after vaccination, which was further confirmed by higher IL-2 production when cytokine concentration was measured as iMFI value (Figure 3). Although IL-2 has little direct effector function, it has the ability to expand effector functions of other T cells [23]. In the linear model of differentiation for CD4^+^ Th1 cells, IL-2^+^ CD4 T cells belong to memory cells and have the potential to differentiate into IFN-*γ*-producing cells after recalling by the relevant antigens [23]. Thus, rBCG186 and rBCG486, because of the incorporation of chimeric antigen Ag856A2, enhance the memory capacity of host to* M. tb* pathogen. However, we did not detect any differences of CD4 T cells between rBCG186-vaccinated and rBCG486-vaccinated mice. This may attribute to the short vaccination time window we chose, or the real differences lies in other functions of T cells which is beyond the scope of the T cell functions currently tested and may need to be further exploited in the future.

Effective and coordinated participation of cytokines contribute to the TB control. Those relevant Th1 cytokines (e.g., TNF-*α* and IFN-*γ*), in a larger extent, function through activation of macrophages [26]. TNF-*α* and IFN-*γ* synergistically inhibit the growth of* M. tb* in macrophages through stimulating the production of reactive nitrogen intermediates (RNIs) [27, 28]. As for RNIs, iNOS is the vital enzyme involved for the production of RNIs [29, 30]. TNF-*α*, IFN-*γ*, and iNOS give proper containment of* M. tb* in the early stage [31]. At later stage of infection when inhibition or killing of* M. tb* is well established, their levels of expression will go down to a reasonable value; otherwise immune-pathological response would happen [32]. rBCGs, especially rBCG486, induced enhanced protection against* M. tb* infection in this study (Figure 4). Consistent with the protective efficacy, the inflammation responses in the infected lungs alleviated greatly in rBCGs-vaccinated mice after infection (Figure 5). When measuring the expression levels of inflammatory molecules, the rBCGs-vaccinated mice also displayed reduced levels of expression which were in accordance with the remissive granulomatous inflammation (Figures 5 and 6).

## Supplementary Material

Supplementary Material contains two figures (Figure S1 and Figure S2). FIGURE S1 depicted the schematic diagram of rBCG186 and rBCG486; FIGURE S2 showed the immunohistochemistry of TNF-*α*, IFN-*γ*, and iNOS in spleens of mice 5 wks post-infection.
