# Supplementary figures and images for: Dose of Incorporated Immunodominant Antigen in Recombinant BCG Impacts Modestly on Th1 Immune Response and Protective Efficiency against Mycobacterium tuberculosis in Mice
Source: J Immunol Res. 2014 Jul 23;2014:196124. doi: 10.1155/2014/196124 (PMC4134796; doi:10.1155/2014/196124)

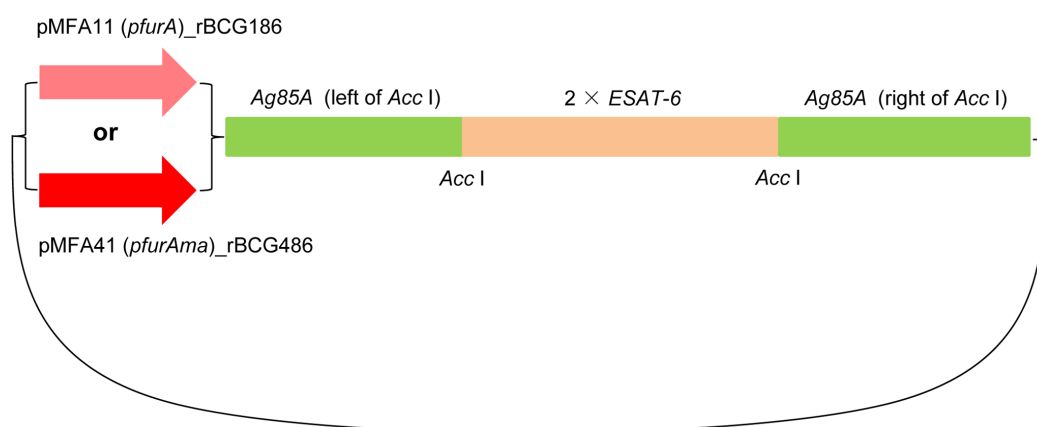

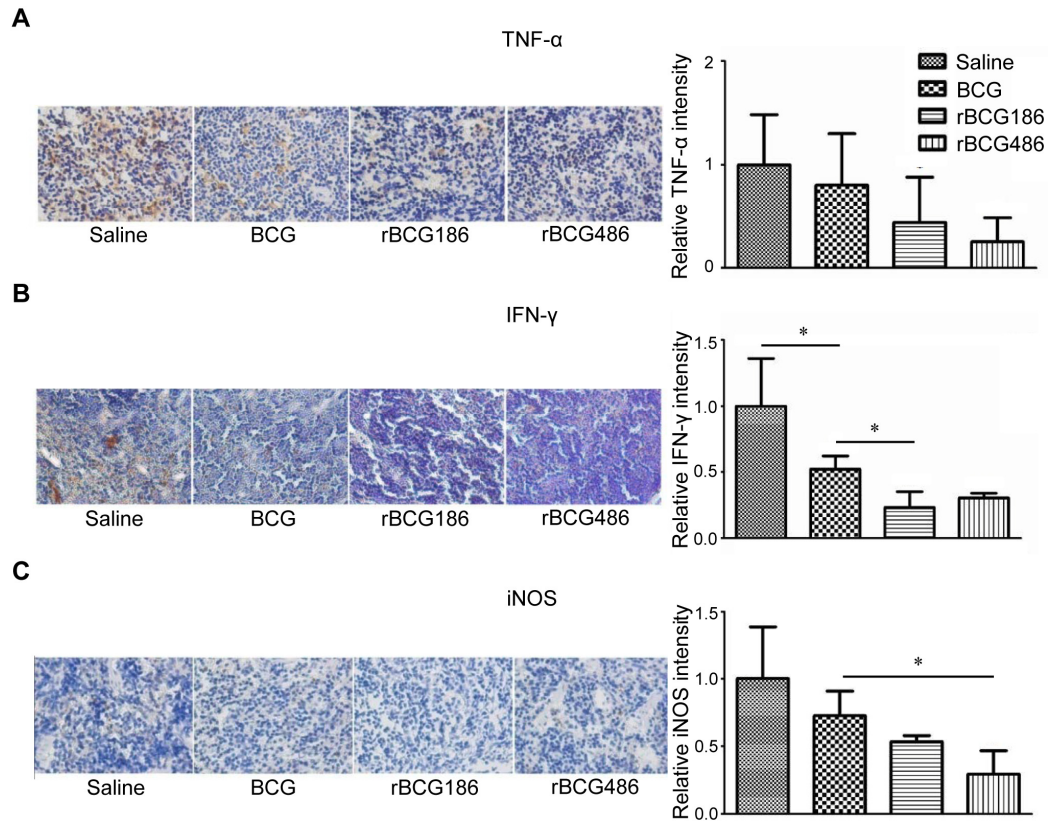

Supplement: Supplementary file 1 — Supplementary Material contains two figures (Figure S1 and Figure S2). FIGURE S1 depicted the schematic diagram of rBCG186 and rBCG486; FIGURE S2 showed the immunohistochemistry of TNF-α, IFN-γ, and iNOS in spleens of mice 5 wks post-infection. [file 196124.f1.pdf]
